# CompareM2 is a genomes-to-report pipeline for comparing microbial genomes

**DOI:** 10.1093/bioinformatics/btaf517

**Published:** 2025-09-15

**Authors:** Carl M Kobel, Velma T E Aho, Ove Øyås, Niels Nørskov-Lauritsen, Ben J Woodcroft, Phillip B Pope

**Affiliations:** Faculty of Biosciences, Norwegian University of Life Sciences, Ås, Norway; Faculty of Biosciences, Norwegian University of Life Sciences, Ås, Norway; Faculty of Biosciences, Norwegian University of Life Sciences, Ås, Norway; Clinical Institute, University of Southern Denmark, Odense, Denmark; Centre for Microbiome Research, School of Biomedical Sciences, Queensland University of Technology (QUT), Translational Research Institute, Woolloongabba, Australia; Faculty of Biosciences, Norwegian University of Life Sciences, Ås, Norway; Centre for Microbiome Research, School of Biomedical Sciences, Queensland University of Technology (QUT), Translational Research Institute, Woolloongabba, Australia; Faculty of Chemistry, Biotechnology and Food Science, Norwegian University of Life Sciences, Ås, Norway

## Abstract

**Summary:**

Here, we present CompareM2, a genomes-to-report pipeline for comparative analysis of bacterial and archaeal genomes derived from isolates and metagenomic assemblies. CompareM2 is easy to install and operate, designed in such a way that the user can install the complete software in one step and launch all analyses on a set of microbial genomes (bacterial and archaeal) in a single action. The central results generated via the CompareM2 workflow are emphasized in a portable dynamic report document.

**Availability and implementation:**

CompareM2 is a free software that is scalable to a range of project sizes, and welcomes modifications and pull requests from the community on its Git repository at https://github.com/cmkobel/comparem2.

## 1 Introduction

Costs are decreasing both for sequencing of microbial genomes and complex microbiomes and for the computational resources necessary to analyze generated reads. This has led to an exponential growth in the number of available isolate genomes and metagenome-assembled genomes (MAGs). Despite this growth, there are limits on the accessibility of software that can analyze the evolutionary relationships and functional characteristics of microbial genomes in order to assess variation of both known and unknown species. Much of the software commonly used to analyze prokaryotic genomes has a high user entry level, requiring advanced skills for complicated installation procedures, debugging dependency issues, and circumventing operating system-specific limitations. This results in a disproportionate amount of time being spent by researchers on setup and technical preparations needed to analyze the sequenced genomic reads rather than biologically relevant analysis of scientific data. These factors define the backdrop that has motivated the conceptualization, development, and application of the CompareM2 genomes-to-report pipeline, which is designed to be an easy-to-install, easy-to-use bioinformatic pipeline that makes extensive analysis and comparison of microbial genomes straightforward.

Another bottleneck in bioinformatics is the interpretation of large output files and visualization of data in an informative manner. CompareM2 produces a graphical report that contains the most important curated results from each of the analyses carried out on the user-specified set of query genomes. This report contains text and figures that explain the significance of the results, which makes it easy to interpret for users with a non-bioinformatics background. While CompareM2 can be used to compare prokaryotic isolate genomes, it also contains tools to analyze bins or MAGs from the sequencing of large microbial communities.

## 2 Methods

### 2.1 Features

**Figure 1.**
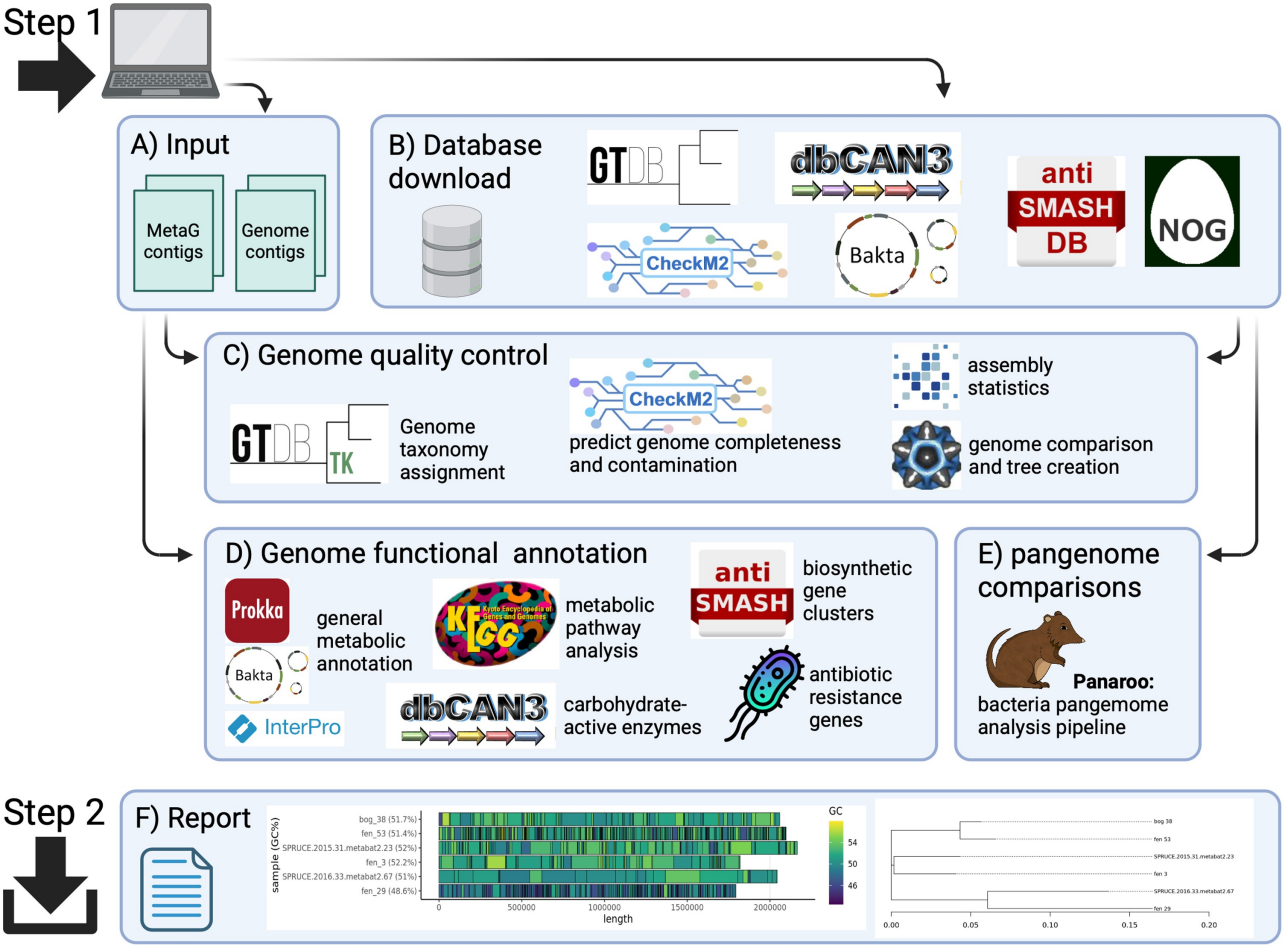
CompareM2 workflow. CompareM2 is designed to enable the user to install the complete software and all the required databases in a single step. Similarly, running all analyses on a set of microbial genomes (bacterial and archaeal) can be launched via a single command line, and the curated results can be downloaded and studied in a dynamically rendered report. Detailed overview of the workflow is available in the Supplementary Information. Created in BioRender. Pope (2025) https://BioRender.com/v13sz83.

The genome is the foundation of any bacterial or archaeal omics study, as it defines the origin of any biological or metabolic phenomenon. One clear example is found in metaproteomics, where protein searches require a highly specific and well-annotated genome database, often derived from MAGs, to match MS/MS spectral data (Andersen *et al.* 2023, Lazear 2023). CompareM2 congregates the most commonly used and community-tested tools to perform prokaryotic genome quality control, gene calling, functional annotation, phylogenetic analysis, and comparison of genomes across the core-pan spectrum (Fig. 1, available as supplementary data at *Bioinformatics* online). Quality control is performed by assembly-stats (sanger-pathogens) and seqkit (Shen *et al.* 2024) which both compute various basic genome statistics such as genome length, count and lengths of contigs, N50, and GC content. CheckM2 (Chklovski *et al.* 2023) is run to compute the completeness and contamination parameters of the input genomes. Subsequently, input genomes can be functionally annotated with Bakta (Schwengers *et al.* 2021) (default) or Prokka (Seemann 2014). As both of these annotators produce results with a similar output structure, it is up to the user to decide which to use for downstream analysis.

Advanced genome annotation is carried out with the following tools: Interproscan (Zdobnov and Apweiler 2001) scans protein signature databases like PFAM, TIGRFAM, KEGG (Kanehisa 2013) and HAMAP; dbCAN (Yin *et al.* 2012) scans carbohydrate active enzymes (CAZymes); Eggnog-mapper (Cantalapiedra *et al.* 2021) provides orthology-based functional annotations; Gapseq (Zimmermann *et al.* 2021) builds gapfilled genome scale metabolic models (GEMs); Antismash (Blin *et al.* 2023) finds biosynthetic gene clusters; and Clusterprofiler (Wu *et al.* 2021) computes a pathway enrichment analysis. For taxonomic assignment of input genomes, GTDB-Tk (Chaumeil *et al.* 2022) uses an alignment of ubiquitous proteins to predict species names. In a clinical setting, the following tools might be useful: AMRFinder (Feldgarden *et al.* 2019) scans for antimicrobial resistance genes and virulence factors, and MLST (Seemann 2025) calls multi-locus sequence types, which is relevant for an initial grouping when tracking transmission and spread of bacteria. In terms of phylogenetic analysis: Mashtree (Katz *et al.* 2019), computes a neighbor-joined tree on the basis of mash distances, whereas Treecluster (Balaban *et al.* 2019) clusters the mashtree tree. Finally, Panaroo (Tonkin-Hill *et al.* 2020) produces a core genome suitable for phylogenetic analysis and defines a pangenome. This core genome is used by the following tools: Fasttree 2 (Price *et al.* 2010) computes a neighbor-joined tree; IQ-TREE 2 (Minh *et al.* 2020) computes a maximum-likelihood tree; and Snp-dists (Seemann *et al.* 2025) computes the pairwise SNP distances.

A major priority of CompareM2 is the ease of installation and use, which is achieved by containerizing all bundled software packages and automatizing the download and setup of databases (Fig. 1). The choice of genomes to input can be any set where there is a comparable feature either within or between species. The number is limited by the computational resources, but the dynamic report is designed for comparing hundreds of genomes. CompareM2 also allows users to add RefSeq or GenBank genomes as references for comparison with their own genomes. The user only needs to specify the relevant accessions when starting the pipeline, and the genomes and their annotations are automatically downloaded and integrated into the downstream analysis.

### 2.2 Software design

CompareM2 is written as a command line program that the user calls with the input genomes that they wish to analyze. It has a text interface where the user can define optional parameters and a single executable that takes care of the overall procedure: First, it checks for presence of the Apptainer runtime and defines reasonable defaults for database directories and configuration files, in case the user has not specified these manually as environment variables. There is also a “passthrough arguments” feature that makes it possible to address any command line argument to any rule in the workflow. (further details in the documentation at https://comparem2.readthedocs.io/en/latest/). One example of a setting that can be defined via the configuration file is whether to optionally submit jobs through a workload manager such as Slurm or PBS, which are typically used on high-performance computing clusters (HPCs). Next, the executable dispatches the main Snakemake pipeline that runs all genomic analyses. This main pipeline automatically installs all necessary software environments and automatically downloads necessary databases, depending on which analyses the user has selected to run. Finally, it dispatches rendering of the dynamic report which contains the results of the main pipeline. This report is dynamic in the sense that it only includes the results which are present, which means that it can be rendered independently of which analyses the user has selected to compute.

Overall, CompareM2 is designed in such a way that the user can install the complete software in a single step. Similarly, running all analyses on a set of microbial genomes (bacterial and archaeal) can be launched in a single action, and the curated results can be downloaded and studied in the dynamically rendered report. The machine requirements are a Linux-compatible OS with a Conda-compatible package manager, e.g. Miniforge, Mamba, or Miniconda. There is nothing standing in the way of running CompareM2 on other operating systems, but many of the included bioinformatic tools are only fully compatible with Linux-like x64-based systems. For a technical description of how CompareM2 is implemented, please see the Methods section (Supplementary Information, available as supplementary data at *Bioinformatics* online).

## 3 Results

The central results generated via the CompareM2 workflow are emphasized in a portable dynamic report document that contains results text and figures (for demo reports, please see https://comparem2.readthedocs.io/en/latest/30%20what%20analyses%20does%20it%20do/#rendered-report). Benchmarking of CompareM2 showed that it is significantly faster than the comparable software Tormes and Bactopia, as its running time scales much better with increasing input size (Supplementary Information, available as supplementary data at *Bioinformatics* online). Notably, running time scaled approximately linearly with a small slope even when increasing the number of input genomes well beyond the number of available cores on the machine. The running time of each pipeline comes down to the time it takes to run each included tool on each sample, so differences between pipelines in terms of running time are determined by how they allocate resources and schedule jobs efficiently in parallel.

The speed of Bactopia is strongly affected by its reads-based approach: If reads are not input by the user—which was not possible in this case because we compared genomes that were assembled using a different pipeline—Bactopia creates artificial reads with ART (Huang *et al.* 2012). This is done in order for Bactopia to be able to compare genomes without reads to genomes with reads. CompareM2 on the other hand is designed to compare genomes without reads and thus does not have to spend computing resources on producing these artificial reads. It should be noted that if the user runs more comparative analyses using the Bactopia Tools extensions, the scalability will be worse since the Bactopia platform does not offer to schedule running of several tools in parallel. While Tormes does not suffer from producing artificial reads, it does fall short on not having a parallel workflow management system. As it runs all samples sequentially, running each tool at a time, it is not competitive on HPCs or multi-core CPUs. Generally, the running time standard deviations are negligible because the relative time differences between the tools are large. The running time was computed on a 64-core workstation (see Methods—Benchmarking, Supplementary Information, available as supplementary data at *Bioinformatics* online). We ran the analysis by allocating 32 cores on this machine. By not using all available cores, we lower the chances that any other component than the CPU is the main bottleneck for computation.

Since both Tormes and Bactopia are designed for different use cases, they might not represent the perfect contenders for a comparison with CompareM2. Nonetheless, to our knowledge, they are the most comparable pipelines that exist today. In the case of Tormes, the comparison highlights the benefit of having a parallel rather than sequential job scheduling setup. In the case of Bactopia, it shows that other pipelines can approach the scalability of CompareM2 but also that having a reads-based approach is not competitive and that comparative analyses can be more integrated into the main pipeline. Also, we want to highlight that Bactopia and Tormes are not the only tools relevant for comparison. As CompareM2 sports many tools for advanced annotation, it also overlaps in use case with more annotation-focused pipelines like DRAM (Shaffer *et al.* 2020).

What is characteristic about CompareM2, is that it is assembly-agnostic: It works strictly downstream of assembling and binning. It is a general-purpose pipeline that does not discriminate between genomes based on how they were assembled. Many other tools include all the steps necessary to turn raw reads into genome representatives and then do varying degrees of biological characterization of these, but raw read-dependent tools were deliberately left out of CompareM2. This is because read mapping, assembling, or even binning are highly dependent on the sequencing technology used and require a highly specialized pipeline for each use case. Next-generation sequencing has matured, and many competitive sequencing platforms exist (sequencing-by-synthesis, single molecule sequencing, etc.). Thus, designing a toolbox that can compare genomes is a very different discipline from designing a toolbox that can assemble these genomes in the first place. Hard-linking these two pipelines together therefore raises the concern that one part will not fit a specific use case. CompareM2 takes a different approach which is to offer a platform where you can compare your genomes regardless of how they were assembled.

## 4 Conclusion

CompareM2 offers an easy-to-install, user-friendly, and efficient genome annotation pipeline. It can be launched using a single command and is scalable to a range of projects, from the annotation of single genomes to comparisons across complex inventories. By using widely adopted and freely available genome tools, CompareM2 performs key annotation steps including genome quality control, gene function prediction, and taxonomic assignment. In addition, comparative analyses like computation of core- and pan-genomes or phylogenetic relations can be executed. We expect that CompareM2 will support the productivity of genome researchers by simplifying and expediting the annotation and comparison of genome-centric data. Further development of CompareM2 will continue with its ongoing adaptation to the community consensus of microbial ecologists. Through benchmarking, we have shown that CompareM2 is highly scalable, allowing analysis of large numbers of input genomes thanks to its underlying parallel job scheduling provided by Snakemake. Via CompareM2 we seek to accelerate and democratize the analysis of genomic assemblies for anyone who has computational resources available—be that on HPCs, a workstation, or even a laptop.

## Supplementary Material

btaf517_Supplementary_Data

## Data Availability

All data used to develop and test CompareM2 is available at https://github.com/cmkobel/CompareM2
