# Supplementary material for: CompareM2 is a genomes-to-report pipeline for comparing microbial genomes
Source: Bioinformatics. 2025 Sep 15;41(9):btaf517. doi: 10.1093/bioinformatics/btaf517 (PMC12466929; doi:10.1093/bioinformatics/btaf517)
Supplement: btaf517_Supplementary_Data [file btaf517_supplementary_data.zip › Kobel et al_supplementary information_cmk.docx]

**Supplementary Information**

## **Benchmarking**

Using two separate MAG catalogues (124 *Prevotella-*affiliated MAGs and 44 *Methanobrevibacter*-affiliated MAGs), we compared CompareM2 to several other pipelines that are designed for overlapping use cases: Nullarbor[^25^](https://www.zotero.org/google-docs/?n24tPj), Tormes[^26^](https://www.zotero.org/google-docs/?LyH5Tf) (stylized TORMES) and Bactopia[^27^](https://www.zotero.org/google-docs/?Jg0K8D) (**Table 1**). Nullarbor and Tormes do assembly and comparison and have a focus on antimicrobial resistance, spread of pathogens, and core genomes relevant for analyzing individual species. They both produce a report document that is similar to what CompareM2 produces. Bactopia does both assembly and comparative analyses, but while it does some comparative analyses in conjunction with assembly, the user must launch individual predefined workflows included in the Bactopia Tools extension to compare between the samples. Bactopia does not have a parallel scheduler for running these comparative tools. While it does not produce a report document, it does have more overlapping tools with CompareM2 when considering the Bactopia Tools extension. Neither Tormes nor Bactopia is designed for analyzing archaea, although many of the tools integrated in these pipelines are applicable for archaeal genomes when care is taken, e.g. core/pan genome reconstruction and phylogenetic analysis, etc. Furthermore, there is a lack of tools to analyze archaea which means that in many cases, researchers may opt to use non-archaeal tools for analysis of these. For this reason, we have opted to compare them to CompareM2, which is designed to analyze both bacteria and archaea. Finally, as none of these tools support the external long-reads based assembly, binning and dereplication pipeline where our MAGs were sourced from, we inputted the finished MAGs as is into these tools. Unfortunately, this was not possible for Nullarbor, as it is not able to run without reads[^28^](https://www.zotero.org/google-docs/?fXFnik). Nonetheless, we have included Nullarbor in **Table 1** for the purpose of a qualitative comparison.

***Table 1:*** *Qualitative comparison of Nullarbor, Tormes, Bactopia and CompareM2.*

|  | **Nullarbor**[**^25^**](https://www.zotero.org/google-docs/?5HaWXj) | **Tormes**[**^26^**](https://www.zotero.org/google-docs/?ljpmbF) | **Bactopia**[**^27^**](https://www.zotero.org/google-docs/?fEfCKt) | **CompareM2** |
| --- | --- | --- | --- | --- |
| **Parallel workflow management (system)** | yes (GNU make) | no | yes (Nextflow) | yes (Snakemake) |
| **Built in compatibility with high performance computing (HPC) workload managers.** | no | no | yes | yes |
| **Assembly-agnostic characterization** | no | no | no | yes |
| **Officially designed for Bacteria and Archaea** | no | no | no | yes |
| **Quality control** | yes | yes | yes | yes |
| **Annotation** | yes | yes | yes | yes |
| **Core/pan genome partitioning** | yes | yes | yes (using Bactopia Tools extension) | yes |
| **Phylogenetics** | yes | no | yes (using Bactopia Tools extension) | yes |
| **Portable visual report document** | yes | yes | no | yes |
| **Automated installation** | yes | yes | yes | yes |
| **Minimal number of steps in installation instructions (after installing Conda)** | NA | 3 | 1 | 1 |
| **Automated database download and setup** | no | yes (no checkpoints) | yes | yes |
| **Conda environment solvable with strict channel priority** | NA | no | yes | yes |
| **Docker compatible containerization** | NA | no | yes | yes |
| **Conda recipe availability (channel)** | yes (bioconda) | no | yes (bioconda) | yes (bioconda) |
| **Age of current release** | Approx. 6 years | Approx. 3 years | Approx. 1 month | Approx. 1 month |
| **License** | GPL-v2 | GPL-v3 | MIT | GPL-v3 |
| **Current version** | 2.0.20191013 | 1.3.0 | 3.0.1 | 2.8.1 |
| **Repository** | [github.com/tseemann/nullarbor](https://github.com/tseemann/nullarbor) | [github.com/nmquijada/tormes](https://github.com/nmquijada/tormes) | [github.com/bactopia/bactopia](https://github.com/bactopia/bactopia) | [github.com/cmkobel/comparem2](https://github.com/cmkobel/assemblycomparator2) |

Tormes has a sequential architecture, which means that it runs one sample at a time and one tool at a time. This is in contrast to CompareM2 and Bactopia, which have a parallel job scheduler where several samples and tools can be run at the same time. CompareM2 inherits this property from Snakemake, on which it is built. Bactopia on the other hand is built on the Nextflow workflow system which in many cases is comparable to Snakemake. Central processing units (CPUs) of computers, whether in laptops, workstations, or HPCs, are seeing an increasing number of physical cores. To take advantage of this, it is necessary for software to have a parallel architecture that can utilize the full potential of the processing resources available. This is especially important on HPCs, where many independent compute nodes can run parallel jobs in a scalable manner.

We compared the running times of CompareM2, Tormes, and Bactopia when scaling up the number of input MAGs to analyze on a single workstation. We considered two different genera: *Methanobrevibacter*, which are archaea from the class Methanobacteria, and *Prevotella*, which are Gram-negative bacteria from the class Bacteroidia. Our MAGs have an average genome size of 2.19 Mb for *Methanobrevibacter* and 3.07 Mb for *Prevotella*. Species prediction and genome sizes are measured on the analyzed MAGs with GTDB-Tk[^14^](https://www.zotero.org/google-docs/?DKnL56) and assembly-stats[^3^](https://www.zotero.org/google-docs/?Ha4d3a) using CompareM2 itself.

Although Bactopia, Tormes, and CompareM2 are designed for overlapping use cases, they are still very different, because they implement different kinds of analyses. In order to make them as comparable as possible, we ran only the analyses with pairwise overlap between CompareM2 and each of the two other tools. This was done using CompareM2’s “until” parameter to specify exactly which rules to run. CompareM2 in “Bactopia mode” includes rules sequence_lengths, assembly_stats, prokka, amrfinder, and mlst, whereas CompareM2 in “Tormes mode” includes rules prokka, amrfinder, assembly_stats, mlst, panaroo, and gtdbtk.


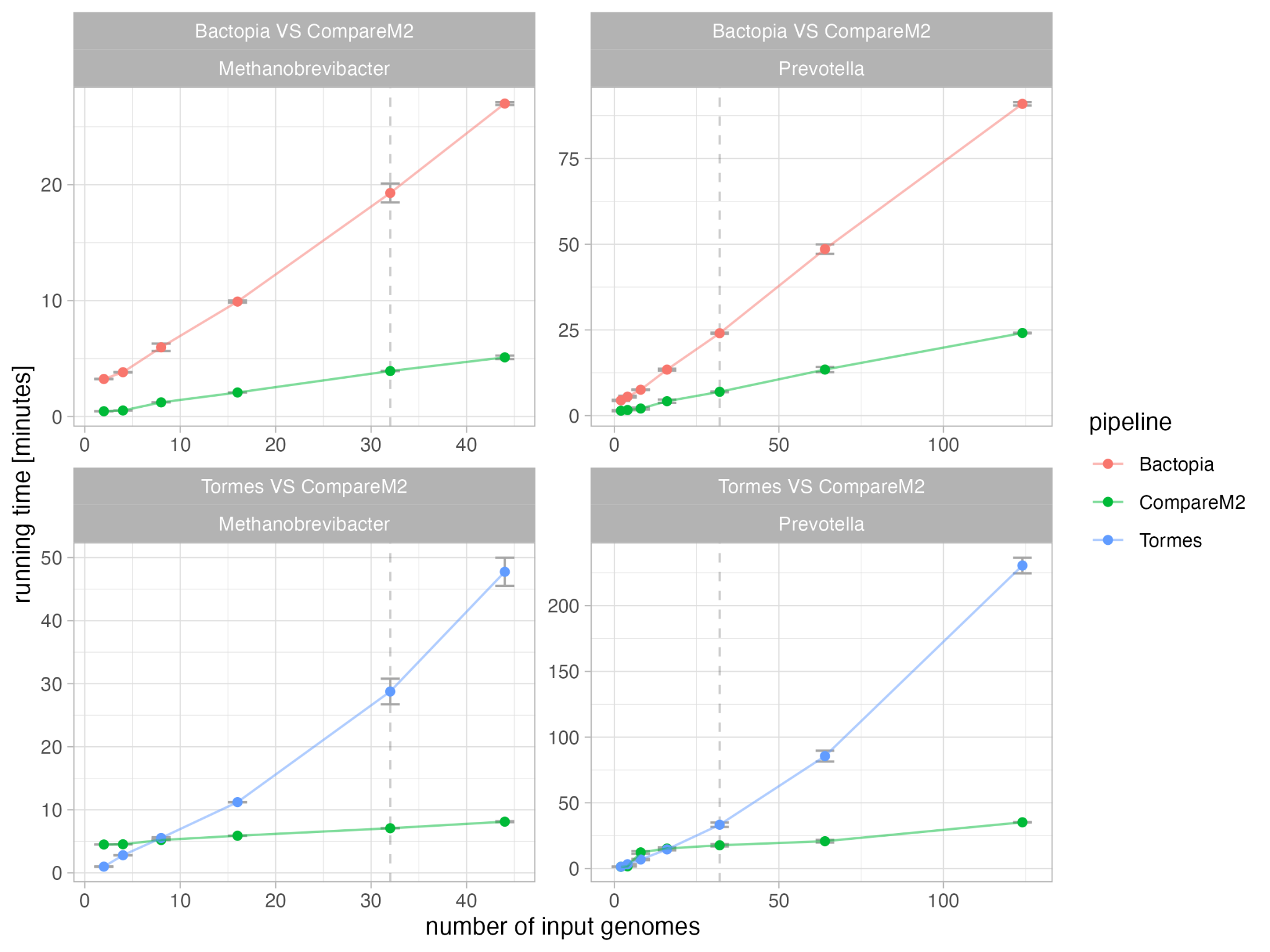


***Fig. 2****:* ***Wall running time analysis comparing CompareM2 to Bactopia and Tormes.*** *In each comparison, CompareM2 was run in a mode where it creates a comparable set of results to the pipeline it is compared to. All analyses ran with 3 replicates, the error bars show means ± the standard deviation of these replicates. A vertical dashed line highlights input size = 32 which is equal to the number of cores used in each benchmark.*

We analyzed the running time of Bactopia, Tormes and CompareM2 when increasing the input size (number of input genomes) (**Fig. 2**). The running times of all tools were approximately linear functions of the input size (time = input size × slope + constant) but with big differences between pipelines in the slope. There are hints of an exponential component in the scaling of running time for Tormes and CompareM2 in Tormes mode, likely because these pipelines construct core genomes, which is a computationally expensive problem where all genes, in the case of Panaroo and Roary, are compared in a pairwise manner[^19,29^](https://www.zotero.org/google-docs/?saRGPB). The running time per genome was generally higher for *Prevotella* than *Methanobrevibacter,* which is expected since *Prevotella* has a slightly larger average genome size, meaning that the total number of genes to be processed is larger.

For running time per number of input genomes, CompareM2 outperformed both Tormes and Bactopia significantly. When analyzing 44 *Methanobrevibacter* MAGs, CompareM2 is 4.1 times faster than Bactopia and 7.2 times faster than Tormes. For 124 *Prevotella MAGs*, CompareM is 3.1 and 7.8 times faster than Bactopia and Tormes, respectively (**Table 2**).

***Table 2****: Wall running time in seconds for analyzing 44 Methanobrevibacter MAGs or 124 Prevotella MAGs. “Factor” denotes how many times slower each tool is compared to the fastest. The fastest tool is marked with an underline (in both cases CompareM2). All numbers are means of three replicates.*

|  | **44 Methanobrevibacter genomes** | | **124 Prevotella genomes** | |
| --- | --- | --- | --- | --- |
|  | **minutes** | **factor** | **minutes** | **factor** |
| **CompareM2** | 6.6 | 1.0 | 29.7 | 1.0 |
| **Bactopia** | 27.0 | 4.1 | 91.0 | 3.1 |
| **Tormes** | 47.7 | 7.2 | 230.5 | 7.8 |

**References**

[25. tseemann/nullarbor: :floppy_disk: ‘Reads to report’ for public health and clinical microbiology. https://github.com/tseemann/nullarbor?tab=readme-ov-file.](https://www.zotero.org/google-docs/?GpbZ40)

[26. Quijada, N. M., Rodríguez-Lázaro, D., Eiros, J. M. & Hernández, M. TORMES: an automated pipeline for whole bacterial genome analysis. *Bioinformatics* **35**, 4207–4212 (2019).](https://www.zotero.org/google-docs/?GpbZ40)

[27. Petit, R. A. & Read, T. D. Bactopia: a Flexible Pipeline for Complete Analysis of Bacterial Genomes. *mSystems* **5**, 10.1128/msystems.00190-20 (2020).](https://www.zotero.org/google-docs/?GpbZ40)

[28. Nullarbor report without reads · Issue #249 · tseemann/nullarbor. https://github.com/tseemann/nullarbor/issues/249.](https://www.zotero.org/google-docs/?GpbZ40)

[29. Page, A. J.](https://www.zotero.org/google-docs/?GpbZ40) *[et al.](https://www.zotero.org/google-docs/?GpbZ40)* [Roary: rapid large-scale prokaryote pan genome analysis. *Bioinformatics* **31**, 3691–3693 (2015).](https://www.zotero.org/google-docs/?GpbZ40)
